# Fermented Soy and Fish Protein Dietary Sources Shape Ileal and Colonic Microbiota, Improving Nutrient Digestibility and Host Health in a Piglet Model

**DOI:** 10.3389/fmicb.2022.911500

**Published:** 2022-06-22

**Authors:** Ying Li, Yunsheng Han, Qingyu Zhao, Chaohua Tang, Junmin Zhang, Yuchang Qin

**Affiliations:** ^1^State Key Laboratory of Animal Nutrition, Institute of Animal Sciences of Chinese Academy of Agricultural Sciences, Beijing, China; ^2^Scientific Observing and Experiment Station of Animal Genetic Resources and Nutrition in North China of Ministry of Agriculture and Rural Affairs, Institute of Animal Science of Chinese Academy of Agricultural Sciences, Beijing, China; ^3^Key Laboratory of Feed Biotechnology, Ministry of Agriculture and Rural Affairs, Feed Research Institute of Chinese Academy of Agricultural Sciences, Beijing, China

**Keywords:** fermented soybean meal, fish meal, growth performance, ileac and colonic microbiota, apparent total tract digestibility, serum immunity

## Abstract

Suitable protein sources are essential requirements for piglet growth and health. Typically, intestinal microbiota co-develops with the host and impact its physiology, which make it more plastic to dietary protein sources at early stages. However, the effects of fermented soybean meal (FSB) and fish meal (FM) on foregut and hindgut microbiota, and their relationship with nutrient digestion and host health remain unclear. In this study, we identified interactions between ileac and colonic microbiota which were reshaped by FSB and FM, and assessed host digestibility and host health in a piglet model. Eighteen weaned piglets (mean weight = 8.58 ± 0.44 kg) were divided into three dietary treatments, with six replicates/treatment. The level of dietary protein was 16%, with FSB, FM, and a mixture of fermented soybean meal and fish meal (MFSM) applied as protein sources. During days 1–14 and 1–28, diets containing MFSM generated higher piglet body weight and average daily gain, but lower feed to weight gain ratios when compared with the FM diet (*P* < 0.05). Piglets in MFSM and FM groups had lower apparent total tract digestibility (ATTD) of crude protein (CP) compared with the FSB group (*P* < 0.05). Serum immunoglobulins (IgM and IgG) in MFSM and FM groups were significantly higher on day 28, but serum cytokines (interleukin-6 and tumor necrosis factor-α) were significantly lower than the FSB group on days 14 and 28 (*P* < 0.05). When compared with FSB and FM groups, dietary MFSM significantly increased colonic acetic acid and butyric acid levels (*P* < 0.05). Compared with the FM and MFSM groups, the FSB diet increased the relative abundance of ileac *Lactobacillus* and *f_Lactobacillaceae*, which were significant positively correlated with CP ATTD (*P* < 0.05). Compared with the FSB group, the relative abundance of *f_Peptostreptococcaceae* and *Romboutsia* in MFSM or FM groups were increased and were significant positively correlated with total carbohydrate (TC) ATTD (*P* < 0.05). Piglets fed FSB had higher α-diversity in colonic microbiota when compared with other groups (*P* < 0.05). The relative abundance of colonic *unidentified_Clostridiales* and *Romboutsia* in MFSM and FSB groups were significantly higher than in the FM group (*P* < 0.05). Dietary MFSM or FM increased the relative abundance of colonic *Streptococcaceae* and *Streptococcus*, but decreased the relative abundance of *Christensenellaceae* when compared with the FSB group (*P* < 0.05). These bacteria showed a significantly positive correlation with serum cytokine and immunoglobulin levels (*P* < 0.05). Therefore, dietary FSB improved CP digestibility by increasing the relative abundance of ileac *f_Lactobacillaceae* and *Lactobacillus*, while dietary MFSM benefited TC digestibility by increasing *f_Peptostreptococcaceae* and *Romboutsia*. Dietary MFSM and FM enhanced immunoglobulin secretion by increasing colonic *f_Streptococcaceae* and *Streptococcus* prevalence, while dietary FSB promoted cytokine production by increasing microbiota diversity and *Romboutsia* and *Christensenellaceae*. Our data provide a theoretical dietary basis for young animals using plant and animal protein sources.

## Introduction

Dietary protein is a fundamental source of amino acids for mammals, and an essential requirement for physiological organ function and neurodevelopment in early life stages. In the gastrointestinal tract, dietary proteins interplay with a variety of substrates. Most are digested as peptides and amino acids by proteinases in the small intestine. Others enter the hindgut to undergo microbial fermentation (Walker et al., 2005; Blachier et al., 2007). High protein levels and poor protein digestibility in diets increase protein influx into the hindgut, cause excess fermentation, and release toxic metabolites which are detrimental to the host (Yao et al., 2016; Zhang and Piao, 2022). Thus, reducing dietary protein levels and optimizing existing protein sources may be effective in reducing abnormal fermentation processes in the hindgut.

In the pig industry, weaned piglets often experience a transition from highly digestible milk to solid protein diets which induce intestinal disorders and cause diarrhea or even death (Hu et al., 2020). The piglet model is suitable to investigate the effects of dietary protein sources on gut microbiota and health as they are susceptible to protein levels and quality (Li et al., 2019b). Fish meal (FM) has long been used as an ideal dietary protein source for animal production as it contains a balanced source of indispensable amino acids, and a rich source of long-chain omega-3 fatty acids, vitamins, and minerals (Zhang et al., 2018a). Soybean meal is derived from plant proteins and is considered an excellent protein source approximately equivalent to animal proteins, and it rates as 1.0 on the protein digestibility corrected amino acid score scale (Hasler and Clare, 2002). Low protein diets with balanced amino acids can reduce diarrhea incidence and maintain intestinal health without affecting piglet performance (Wang et al., 2018). Reducing crude protein levels by 3% could shift colonic microbiota in pigs, decrease the relative abundance of *Streptococcus* but increase the relative abundance of *Sarcina*, *Coprococcus*, and *Peptostreptococcaceae* (Zhou et al., 2016). Fermentation improves soybean meal quality by decreasing anti-nutritional factors, such as protease inhibitors, and enhancing free amino acid and small peptide levels (Seo and Cho, 2016; Zhang et al., 2016). Also, fermented soybean meal (FSB) administration decreases the incidence of diarrhea in pigs by inhibiting intestinal pathogen colonization (Kiers et al., 2010).

The gastrointestinal tract contains a dense, dynamic, and highly complex microbial community, equating to appropriately 10^14^ microbes and comprising thousands of individual species (Collins et al., 2012). Swine intestinal microbiota co-develop with the host and play important roles in nutrient digestion, absorption, metabolism, and intestinal health protection (Li et al., 2019b). Microbiota composition is in flux, but over time, diversity increases and converges toward an adult-like microbiota, with features in increasing overall number of taxa and functional genes (Koenig et al., 2011; Rodrguez et al., 2015). During early life stages, this microbial colonization period appears critical for both current- and whole-life health as the microbiota are more plastic and dynamic than in adults. Generally, the intestinal microbiota are resilient to changes in dietary protein sources (Rist et al., 2013). However, little is known about the effects of FSB and FM on host foregut and hindgut microbiota, while their relationship with nutrient digestion and host health remains unclear.

Therefore, we hypothesized that diets containing FSB and FM could alter ileal and colonic microbiota and contribute to host digestibility and health in early life. To this end, we used a piglet model to investigate dietary effects on growth performance and ileal and colonic microbial structures, and to understand the relationship between microbiota, nutrient digestibility, and serum immunity. Our data provide a theoretical dietary basis for young animals using different protein sources for overall health.

## Materials and Methods

### Ethical Considerations

This study was conducted in accordance with the “Guidelines on Welfare and Ethical Review for Laboratory Animals” (GB/T 35892-2018), and it was approved by the Institutional Animal Care and Use Committee of the Institute of Animal Science of the Chinese Academy of Agricultural Sciences, Beijing, China (IAS2019-25).

### Materials

High-quality FSB was selected based on our previous study (Li et al., 2019c). Firstly, three FSB samples were selected from ten representative samples, based on the evaluation of crude protein (CP), amino acids, acid soluble proteins, and anti-nutrient factors, including glycinin, β-conglycinin, and trypsin inhibitors. The highest quality FSB was then selected based on dry matter (DM) and CP digestibility, and *in vitro* amino acid evaluation. FSB was fermented using a combination of *Bacillus subtilis*, *Lactobacillus plantarum*, and *Saccharomyces cerevisiae* at ≥10^9^ CFU/mL, ≥C10^10^ CFU/mL, and ≥10^9^ CFU/mL concentrations, respectively. Also, imported fishmeal (Peru) containing 68.5% CP was selected as a high-quality FM source.

### Animals, Diets, and Experimental Design

Eighteen crossbred male weaned piglets (Duroc × Landrace × Yorkshire, weaned at 28 days), with an average body weight (BW = 8.58 ± 0.44 kg), were randomly allocated to three groups, consisting of six replicates, with one piglet/replicate. FSB (*n* = 6), FM (*n* = 6), and a mixture of fermented soybean meal and fish meal (MFSM, *n* = 6) were used as protein sources. To meet National Research Council (2012) nutrient requirements for 7–11 kg pigs, a non-medicated basal diet in mashed form was formulated, with CP levels at 16% in accordance with a previous study (Chen et al., 2018; Table 1). All diets contained equal standardized ileal digestible (SID) lysine, methionine, threonine, tryptophan, leucine, isoleucine, and valine, using added crystalline amino acids. Piglets were housed alone in one pen (1.8 m × 0.8 m) with a hard plastic, fully-slotted floor. Adjacent pens were separated by a closed baffle. Piglets were housed in an environmentally controlled room (28–30 and 25–28°C between days 1–14 and 15–28, respectively). The study lasted 28 days, during which time meals were provided twice daily at 08:00 and 16:00 h, and pigs had *ad libitum* access to food and water.

**TABLE 1:** Ingredients and chemical composition of experimental diets (as-fed basis).

Ingredient (%)	Diet treatments^§^		
	FSB	FM	MFSM
Corn	65.27	76.77	69.18
Fermented soybean meal	23.50	0.00	15.25
Fish meal	0.00	14.00	5.00
Soybean oil	4.50	4.50	4.50
Dicalcium phosphate	1.50	0.20	1.00
Limestone	0.60	0.10	0.55
Sodium chloride	0.27	0.07	0.20
Glucose	2.00	2.00	2.00
L-Lysine-HCl	0.69	0.61	0.66
DL-Methionine	0.20	0.04	0.14
L-Threonine	0.28	0.30	0.28
L-Tryptophan	0.07	0.10	0.08
L-Leucine	0.00	0.14	0.02
L-Isoleucine	0.13	0.22	0.16
L-Valine	0.28	0.25	0.27
Chromium oxide	0.20	0.20	0.20
Vitamin and mineral premix^†^	0.50	0.50	0.50
Total	100.00	100.00	100.00
**Nutrient composition (%)^‡^**			
Digestible energy (MJ/kg)	14.59	14.77	14.65
Crude protein	16.21	16.27	16.28
Calcium	0.75	0.75	0.79
Total phosphorus	0.66	0.66	0.65
Available phosphorus	0.39	0.48	0.42
SID* Lysine	1.35	1.35	1.35
SID* Methionine	0.39	0.39	0.39
SID* Threonine	0.79	0.79	0.79
SID* Tryptophan	0.22	0.22	0.22
SID* Leucine	1.35	1.35	1.35
SID* Isoleucine	0.69	0.69	0.69
SID* Valine	0.86	0.86	0.86

*^†^A vitamin-mineral premix provided the following nutrients per kg of diet: Vitamin A, 2200 IU; Vitamin D3, 220 IU; Vitamin E, 16 IU; Vitamin K3, 0.5 mg; Vitamin B12, 0.02 mg; Riboflavin, 4 mg; Niacin, 30 mg; Pantothenic acid, 12 mg; Choline chloride, 600 mg; Folic acid, 0.3 mg; Vitamin B1, 1.5 mg; Vitamin B6, 7 mg; Biotin, 0.08 mg; Zn, 100 mg; Mn, 4 mg, Fe, 105 mg; Cu, 6 mg; I, 0.14 mg; Se, 0.3 mg.*

*^‡^Nutrient levels were calculated.*

*SID, standardized ileal digestible.

*^§^FSB, fermented soybean meal diet; FM, fish meal; MFSM, mixture of fermented soybean meal and fish meal diet.*

### Sample Collection and Measurements

#### Growth Performance

Taking each piglet as one unit, BW was recorded at study commencement and every fortnight. The feed intake of each piglet was recorded daily using an electronic feeding system (MXCD-15B, Yangzhou, China). Then, average daily feed intake (ADFI), average daily gain (ADG), and feed to weight gain ratios (F/G) were calculated between days 1–14, 15–28, and 1–28.

#### Apparent Total Tract Digestibility

Representative 1 kg feed samples were collected at study end and stored at 4°C. Between days 25 and 28, approximately 200 g fresh feces from each pen was collected twice a day at 7:00 and 16:00, and immediately frozen at −20°C. Feces was pooled by pen, dried at 65°C for 72 h, and ground down to pass through a 1 mm sieve for analysis. DM, CP, ether extract (EE), and ash in feed and fecal samples were measured according to the Association of Official Analytical Chemists (AOAC, 2012). Chromium (Cr) levels in feed and feces were measured using an atomic absorption spectrophotometer (AAS) (TAS-990super, Beijing, China) based on a method by Williams et al. (1962). In addition, organic matter (OM) and total carbohydrates (TCs) were calculated using the following equations: OM = 1 − ash and TC = DM − CP − EE − ash (Gerritsen et al., 2010). ATTD_nutrient_ = 1 − (Cr_diet_ × Nutrient_feces_)/(Cr_feces_ × Nutrient_diet_) (Long et al., 2017).

#### Serum Immune Globulin and Inflammatory Cytokines

On the morning of days 14 and 28, after fasting overnight, 5 mL blood samples were collected from piglets via jugular vein puncture into a vacutainer. After 2 h, bloods were centrifuged at 3,000 × *g* at 4°C for 10 min to recover serum, and stored at −20°C. Serum immunoglobulin A (IgA), IgG, IgM, interleukin-1β (IL-1β), IL-6, and tumor necrosis factor-α (TNF-α) levels were determined using enzyme-linked immunosorbent assays (Shanghai Enzyme-linked Biotechnology, Co., Ltd., Shanghai, China) following manufacturer’s instructions.

#### Ileal and Colonic Microbiota

After fasting overnight on day 28, piglets were humanely slaughtered 5 min after anesthetic injection. Ileal and colonic chyme was collected into 5 mL centrifuge tubes, snap frozen in liquid nitrogen, and stored at −80°C. Bacterial genomic DNA was extracted from ileal and colonic chyme samples (Qiagen DNA stool mini kit, Hilden, Germany). DNA quantity and quality were assessed by NanoDrop 2000 spectrophotometer (Thermo Fisher Scientific, Waltham, MA United States) and 1% agarose gels, respectively. The V3–V4 hypervariable region of *16S rRNA* was amplified using specific primers (forward 5′-ACTCCTACGGGAGGCAGCA-3′ and reverse 5′-GGACTACHVGGGTWTCTAAT-3′) which contained barcodes. Polymerase chain reaction (PCR) was conducted in a total volume of 20 μL, including 1 × FastPfu buffer, 250 μM dNTP, 0.2 μM each primer, 1 U FastPfu polymerase (Beijing TransGen Biotech, Beijing, China), and 10 ng template DNA. Thermal cycling parameters: initial denaturation at 98°C for 1 min, followed by 30 cycles of 98°C for 10 s, annealing at 50°C for 30 s, elongation at 72°C for 30 s, and a final extension at 72°C for 5 min. PCR products were electrophoresed on 2% agarose gels and purified using a Qiagen gel extraction kit (Qiagen, Hilden, Germany). Sequencing libraries were constructed using TruSeq^®^ DNA PCR-Free Sample Preparation Kit (Illumina, California, CA, United States) according to manufacturer’s recommendations, and index codes were added. Library quality was assessed using the Qubit V.2.0 Fluorometer (Thermo Fisher Scientific, Waltham, MA United States). Qualified DNA libraries were loaded into a NovaSeq platform, capable of 2 × 250 bp paired-end sequencing (Novogene, Beijing, China).

#### Biodiversity Analysis

Paired-end reads were generated and merged using FLASH software (V1.2.7)^1^. Operational taxonomic units with 97% identity were gathered using Uparse (ver. 7.1)^2^. Taxonomic annotations were performed using the Mothur algorithm (70% confidence) in the Silva Database^3^. Alpha-diversity was analyzed using Chao 1^4^, ACE^5^, Shannon^6^, and Simpson^7^ indices. Beta-diversity was visualized using Non-Metric Multi-Dimensional Scaling (NMDS) plots combined with Bray-Curtis distances.

#### Statistical Analysis

Data were analyzed using one-way analysis of variance using comparative averages in SPSS 22.0 (IBM Corp., Armonk, NY, United States). Differences between treatment means for growth performance, ATTD, and serum immune indices were analyzed using Duncan’s multiple-range and least significant difference *post hoc* tests. Data were expressed as the mean ± standard deviation (SD). Correlations between differential gut microbiota and immune indices, and corresponding *P*-values were estimated using Spearman correlation analyzes using gplot and psych packages, respectively. *P*-values < 0.05 (*) were statistically significant and *P*-values < 0.01 (^**^) were extremely significant.

## Results

### Growth Performance

Dietary MFSM and FSB generated significantly higher piglet BWs on day 14 and ADG between days 1–14 (*P* < 0.05; Table 2). Dietary MFSM generated significantly higher piglet BWs on day 28 and ADG between days 1–28 (*P* < 0.05) when compared to FM animals. Between days 1–14, ADFI in the FSB group was significantly higher than the FM group (*P* < 0.05). Also, when compared to the FM group, piglets in FSB and MFSM groups had significantly lower F/G ratios between days 1–14 (*P* < 0.05), and piglets in the MFSM group had significantly lower F/G ratios between days 1–28 (*P* < 0.05).

**TABLE 2:** The effects of different protein dietary sources on weaned piglet growth and development.

Item	Dietary treatments^1^			*P*-value
	FSB	FM	MFSM	
**Body weight (kg)**				
Initial	8.47 ± 0.47	8.65 ± 0.46	8.61 ± 0.47	0.78
Day 14	12.13 ± 0.54^a^	11.19 ± 0.86^b^	12.32 ± 0.23^a^	0.01
Day 28	18.61 ± 1.37^ab^	17.48 ± 1.74^b^	19.89 ± 1.20^a^	0.04
**Day 1–14**				
ADFI (g)^2^	446.98 ± 55.53^a^	367.29 ± 38.19^b^	410.75 ± 42.98^ab^	0.03
ADG (g)^2^	228.55 ± 39.75^a^	158.75 ± 36.27^b^	232.25 ± 32.05^a^	0.01
F/G^2^	1.99 ± 0.33^b^	2.38 ± 0.41^a^	1.78 ± 0.07^b^	0.01
**Day 15–28**				
ADFI (g)	719.94 ± 98.35	730.73 ± 68.27	762.88 ± 73.37	0.64
ADG (g)	415.64 ± 76.09	393.13 ± 69.44	472.88 ± 66.2	0.17
F/G	1.75 ± 0.18	1.89 ± 0.26	1.62 ± 0.07	0.08
**Day 1–28**				
ADFI (g)	581.58 ± 56.25	549.01 ± 41.84	586.81 ± 58.05	0.42
ADG (g)	320.8 ± 51.1^ab^	275.94 ± 42.14^b^	352.56 ± 48.51^a^	0.04
F/G	1.84 ± 0.23^ab^	2.01 ± 0.17^a^	1.68 ± 0.06^b^	0.01

*^a,b^Different superscript letters in a row indicate a significant difference (P < 0.05).*

*^1^FSB, fermented soybean meal; FM, fish meal; MFSM, mixture of fermented soybean meal and fish meal.*

*^2^ADFI, average daily feed intake; ADG, average daily gain; F/G, feed to weight gain ratio.*

*^3^Data were the mean of six replicates with one piglet each.*

### Apparent Total Tract Digestibility

Apparent total tract digestibility (ATTD) of DM and TC were not influenced by dietary protein sources (*P* > 0.05; Table 3). FSB-fed piglets had significantly higher CP ATTD than other groups (*P* < 0.05), whereas FSB and FM piglets had significantly higher OM ATTD than MFSM animals (*P* < 0.05). The EE ATTD in FM animals was significantly higher than MFSM and FSB groups (*P* < 0.05), and EE ATTD in the FSB group was significantly higher than the MFSM group (*P* < 0.05).

**TABLE 3:** The effects of different protein dietary sources on nutrient apparent total tract digestibility (ATTD) in weaned pigs.

Item (%)	Dietary treatments^1^			*P*-value
	FSB	FM	MFSM	
DM^2^	87.82 ± 0.36	88.24 ± 0.36	87.34 ± 0.60	0.06
CP^2^	85.39 ± 0.77^a^	82.29 ± 0.87^b^	81.7 ± 0.71^b^	<0.01
EE^2^	74.63 ± 1.14^b^	78.94 ± 2.29^a^	71.97 ± 0.47^c^	<0.01
OM^2^	89.98 ± 0.33^a^	89.91 ± 0.32^a^	89.24 ± 0.45^b^	0.04
TC^2^	92.08 ± 0.21	92.52 ± 0.29	92.12 ± 0.48	0.19

*^a–c^Different superscript letters in a row indicate a significant difference (P < 0.05).*

*^1^FSB, fermented soybean meal; FM, fish meal; MFSM, mixture of fermented soybean meal and fish meal.*

*^2^DM, dry matter; CP, crude protein; EE, ether extract; OM, organic matter; TC, total carbohydrate.*

*^3^Data were the mean of four replicates with one piglet each.*

### Serum Immune Globulins and Inflammatory Cytokines

On day 14, in MFSM and FM groups, serum IgA levels were higher (*P* < 0.05) and serum IL-6 and TNF-α levels were lower (*P* < 0.05) than the FSB group (Table 4). Serum IgG levels in the FM group were significantly increased and serum IL-1β levels in the MFSM group significantly decreased when compared with the FSB group (*P* < 0.05). On day 28, when compared with the FSB group, MFSM and FM piglets had significantly higher serum IgG and IgM levels (*P* < 0.05) and lower serum IL-1β, IL-6, and TNF-α levels (*P* < 0.05). Piglets in the MFSM group had higher serum IgA levels than FSB and FM groups, and higher serum IgM levels than the FM group (*P* < 0.05).

**TABLE 4:** The effects of different protein dietary sources on serum immunity in weaned piglets.

Item	Dietary treatments^1^			*P*-value
	FSB	FM	MFSM	
**14 days**				
IgA^2^ (μg/mL)	70.22 ± 13.68^b^	91.00 ± 17.60^a^	100.05 ± 9.25^a^	0.01
IgG^2^ (mg/mL)	5.41 ± 0.77^b^	6.33 ± 0.50^a^	6.12 ± 0.57^ab^	0.05
IgM^2^ mg/mL)	2.05 ± 0.22	2.18 ± 0.30	2.34 ± 0.41	0.32
IL-1β^2^ (pg/mL)	119.24 ± 10.44^a^	107.00 ± 15.87^ab^	99.37 ± 14.65^b^	0.07
IL-6^2^ (pg/mL)	290.57 ± 14.86^a^	251.38 ± 18.46^b^	247.31 ± 10.58^b^	<0.01
TNF-α^2^ (pg/mL)	62.60 ± 3.08^a^	53.21 ± 5.22^b^	53.93 ± 4.04^b^	<0.01
**28 days**				
IgA (μg/mL)	88.19 ± 14.29^b^	100.53 ± 13.47^b^	127.50 ± 13.54^a^	<0.01
IgG (mg/mL)	5.67 ± 0.64^b^	6.86 ± 0.59^a^	7.37 ± 0.57^a^	<0.01
IgM (mg/mL)	1.90 ± 0.27^c^	2.68 ± 0.23^b^	3.22 ± 0.22^a^	<0.01
IL-1β (pg/mL)	123.59 ± 10.31^a^	90.25 ± 17.15^b^	79.97 ± 15.89^b^	<0.01
IL-6 (pg/mL)	279.70 ± 19.13^a^	219.23 ± 20.16^b^	217.13 ± 20.06^b^	<0.01
TNF-α (pg/mL)	58.00 ± 3.57^a^	49.10 ± 5.58^b^	44.82 ± 5.81^b^	<0.01

*^a–c^Different superscript letters in a row indicate a significant difference (P < 0.05).*

*^1^FSB, fermented soybean meal; FM, fish meal; MFSM, mixture of fermented soybean meal and fish meal.*

*^2^IgA, immunoglobulin A; IgG, immunoglobulin G; IgM, immunoglobulin M; IL-1β, interleukin-1β; IL-6, interleukin-6; TNF-α, tumor necrosis factor-α.*

*^3^Data were the mean of six replicates with one piglet each.*

### Microbiota Diversity in the Ileum and Colon

**FIGURE 1:**
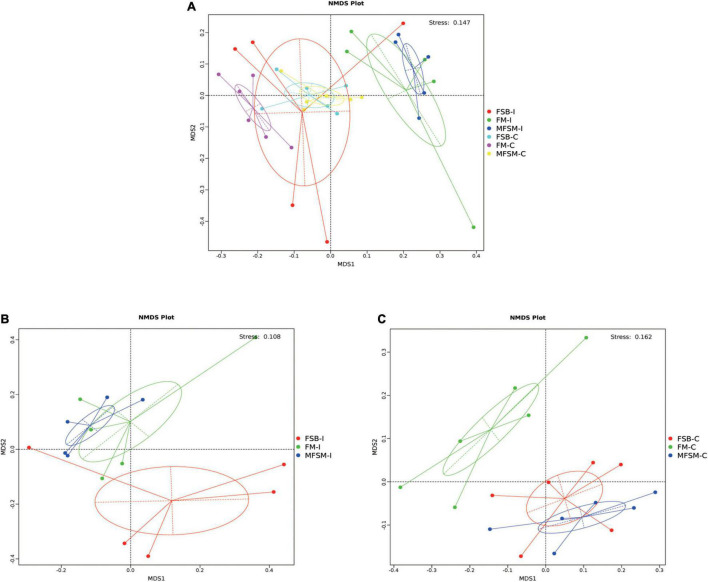
Different protein sources induced microbiota shifts. **(A)** Non-metric multidimensional scaling analysis based on weighted Unifrac distances identified a separation between ileal and colonic segments. **(B,C)** A separation between different groups in ileum and colon. FSB-I, fermented soybean meal-ileal group; FM-I, fish meal-ileal group; MFSM-I, mixture of fermented soybean meal and fish meal-ileal group; FSB-C, fermented soybean meal-colonic group; FM-C, fish meal-colonic group; MFSM-C, mixture of fermented soybean meal and fish meal-colonic group. Data were presented as the mean of five (ileum) and six replicates (colon) with one piglet each.

In total, 2,294,708 effective sequences were generated from piglet gut microbiota samples, with an average 69,537 sequences/sample. Alpha-diversity analyzes showed varied community richness between groups in terms of colonic bacterial communities; the Chao 1 index in FSB animals was higher than FM and MFSM groups, and both Observed_species and ACE indices in the FSB group were higher than those in the MFSM group (*P* < 0.05; Table 5). However, no significant differences in alpha diversity were observed in the ileum (*P* > 0.05). Beta-diversity NMDS analysis based on Bray-Curtis distances showed microbial communities were well separated between the ileum and colon (Figure 1A), and among groups in the ileum (Figure 1B) and colon (Figure 1C). Thus, piglet gut microbiota composition was shaped by different protein sources.

**TABLE 5:** The effects of different protein dietary sources on bacterial α-diversity indices in piglets.

Item (%)	Dietary treatments^1^			*P*-value
	FSB	FM	MFSM	
**Ileum**				
Observed_species	502 ± 91	484 ± 91	457 ± 153	0.57
Shannon	3.86 ± 0.62	3.31 ± 0.41	3.28 ± 0.79	0.19
Simpson	0.791 ± 0.141	0.777 ± 0.054	0.708 ± 0.149	0.33
Chao 1	577 ± 121	568 ± 101	528 ± 158	0.58
ACE	597 ± 122	590 ± 114	541 ± 159	0.54
**Colon**				
Observed_species	698 ± 61^a^	646 ± 49^ab^	617 ± 52^b^	0.05
Shannon	5.65 ± 0.57	5.47 ± 0.72	5.45 ± 0.48	0.59
Simpson	0.924 ± 0.040	0.912 ± 0.063	0.932 ± 0.025	0.48
Chao 1	768 ± 62^a^	702 ± 40^b^	677 ± 54^b^	0.03
ACE	773 ± 62^a^	709 ± 40^ab^	687 ± 52^b^	0.03

*^a,b^ Different superscript letters in a row indicate a significant difference (P < 0.05).*

*^1^FSB, fermented soybean meal; FM, fish meal; MFSM, mixture of fermented soybean meal and fish meal.*

*^2^Data were the mean of five replicates (ileum) and six replicates (colon) with one piglet each.*

### Ileal and Colonic Bacterial Community Structures

**FIGURE 2:**
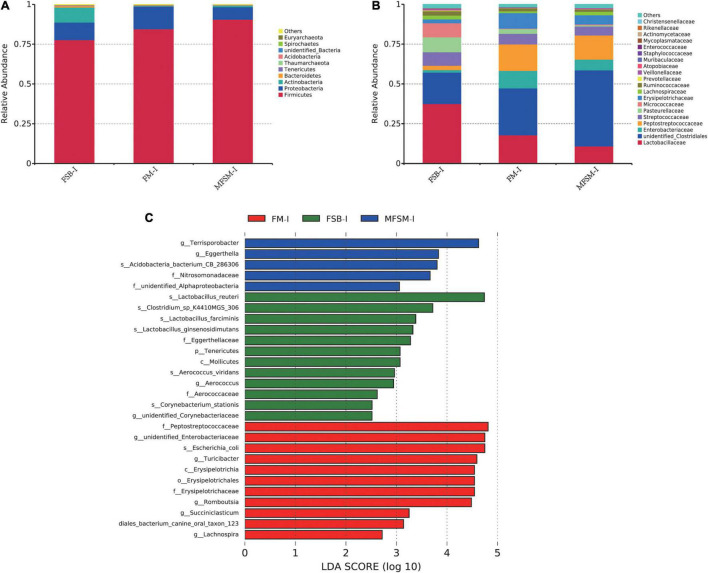
**(A,B)** The effects of different protein sources on bacterial composition in the ileum, at phylum and family levels. **(C)** Taxa enrichment based on linear discriminant analysis effect size (LEfSe) analysis identified significant differences in microbial communities between groups. Bacterial taxa with a logarithmic LDA score > 2.5 were selected as biomarker taxa. FSB-I, fermented soybean meal-ileal group; FM-I, fish meal-ileal group; MFSM-I, mixture of fermented soybean meal and fish meal-ileal group. Data were presented as the mean of five replicates with one piglet each.

In the ileum, *Firmicutes*, *Proteobacteria*, and *Actinobacteria* were the three major bacterial phyla accounting for >95% of total ileal bacterial communities (Figure 2A). The relative abundance of *Firmicutes* in the MFSM-ileal (MFSM-I) group was 90.49%, and was higher than the 84.54 and 77.64% levels in FM-ileal (FM-I) and FSB-ileal (FSB-I) groups (*P* > 0.05), respectively. The relative abundance of *Proteobacteria* in the MFSM-I group was 7.87%, and was lower than the 14.17 and 11.08% levels in FM-I and FSB-I groups (*P* > 0.05), respectively. The relative abundance of *Actinobacteria* in FM-I and MFSM-I groups was 0.43 and 0.94%, respectively, but lower than the 9.40% in the FSB-I group (*P* > 0.05).

At the family level, the relative abundance of *Lactobacillaceae* decreased from 37.54 to 17.85 and 10.85% (FSB-I group to FM-I and MFSM-I groups, respectively), while *unidentified_Clostridiales* levels were opposite, with increases from 19.72 to 29.46 and 47.79%, respectively (*P* > 0.05; Figure 2B). When compared with FSB-I and MFSM-I groups, the FM-I group showed an increased relative abundance of *Enterobacteriaceae*, from 1.55 and 6.77 to 11.01%, respectively (*P* > 0.05). The relative abundance of *Peptostreptococcaceae* in MFSM-I and FM-I groups was 16.62 and 15.13% higher, respectively, than the 2.68% in the FSB-I group (*P* > 0.05). The relative abundance of *Pasteurellaceae* was lowest in the MFSM-I group at 0.77%, in comparison with FSB-I and FM-I groups at 9.36 and 2.96%, respectively (*P* > 0.05). Linear discriminant analysis effect size showed that FM-I piglets had a higher relative abundance of the genera, *unidentified_Enterobacteriaceae*, *Turicibacter*, *Romboutsia*, *Succiniclasticum*, and *Lachnospira* when compared with FSB-I and MFSM-I piglets (Figure 2C). *Lactobacillus_reuteri*, *Lactobacillus_farciminis*, *Lactobacillus_ginsenosidimutans*, and *Clostridium_sp_K4410MGS_306* were dominant bacteria in the FSB-I group when compared with the other groups, whereas the relative abundance of *Terrisporobacter* and *Eggerthella* was significantly increased in the MFSM-I group when compared with FM-I and FSB-I groups.

**FIGURE 3:**
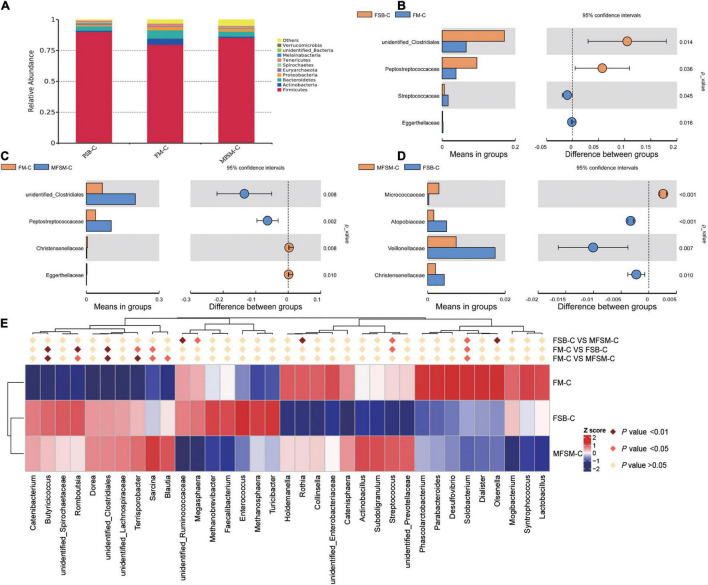
The effects of different protein sources on bacterial compositions in the colon, at phylum, family, and genus levels. **(A)** Distribution of colonic bacteria at the phylum level. **(B–D)** Statistical analysis of differences at the family level; t-tests were used to test for significant differences; *P* < 0.05 indicates a significant difference. **(E)** In the top 35 genera, Metastat was used to test for significant differences in genera relative abundance; deep pink diamonds indicate *P* < 0.05 and dark pink diamonds indicate *P* < 0.01 between two groups. FSB-C, fermented soybean meal-colonic group; FM-C, fish meal-colonic group; MFSM-C, mixture of fermented soybean meal and fish meal-colonic group. Data were presented as the mean of six replicates with one piglet each.

In the colon, *Firmicutes* was the most predominant phylum, with a relative abundance of 90.04, 79.74, and 85.36%, respectively, in FSB-colonic (FSB-C), FM-colonic (FM-C), and MFSM-colonic (MFSM-C) groups (Figure 3A). The relative abundance of *Actinobacteria* (5.02%) and *Bacteroidetes* (6.82%) in the FM-C group was higher than FSB-C (1.12 and 3.55%, respectively) and MFSM-C (1.04 and 4.01%, respectively) groups (*P* < 0.05).

At the family level, when compared with the FM-C group, both FSB-C and MFSM-C groups had a significantly increased relative abundance of *unidentified_Clostridiales* and *Peptostreptococcaceae* (*P* < 0.05; Figures 3B–D). The relative abundance of *Streptococcaceae* in the FM-C group was higher, and the relative abundance of *Atopobiaceae*, *Veillonellaceae*, and *Christensenellaceae* in the MFSM-C group was lower than the FSB-C group (*P* < 0.05).

Of the 35 most dominant colonic genera, the relative abundance of *Butyricicoccus*, *Romboutsia*, *unidentified_Clostridiales*, *Terrisporobacter*, and *Sarcina* in MFSM-C and FSB-C groups was significantly higher than the FM-C group (*P* < 0.05; Figure 3E). When compared with the FSB-C group, the MFSM-C group had a significantly decreased relative abundance of *unidentified_Ruminococcaceae*, *Olsenella*, and *Megasphaera* (*P* < 0.05), but a significantly increased relative abundance of *Rothia* (*P* < 0.05). The relative abundance of *Streptococcus* and *Solobacterium* in the FSB-C group was lower than MFSM-C and FM-C groups (*P* < 0.05), while the relative abundance of *Solobacterium* in the FSB-C group was higher than the MFSM-C group (*P* < 0.05).

### Correlations Between Gut Microbiota and Apparent Total Tract Digestibility or Immune Parameters

**FIGURE 4:**
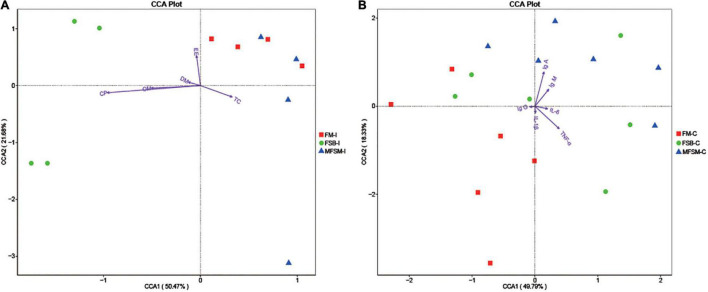
**(A,B)** The Canonical correspondence analyses (CCA) of ileal segments between nutrient apparent total tract digestibility (ATTD) and the first 35 taxa at genus level, and the CCA of colonic segments between immune indices and the first 35 taxa at genus level. “” indicates the first 35 bacterial taxa in each group. Arrows indicate nutrient ATTD and immune indices, respectively. The closer the pendulum is to the arrow, the higher the positive correlation between bacteria and the index, and vice versa. If the angle between the arrows is acute, a positive correlation exists between measured indices, and the converse indicates a negative correlation. FSB-I, fermented soybean meal-ileal group; FM-I, fish meal-ileal group; MFSM-I, mixture of fermented soybean meal and fish meal-ileal group; FSB-C, fermented soybean meal-colonic group; FM-C, fish meal-colonic group; MFSM-C, mixture of fermented soybean meal and fish meal-colonic group. DM, dry matter; CP, crude protein; EE, ether extract; OM, organic matter; TC, total carbohydrate; IgA, immunoglobulin A; IgG, immunoglobulin G; IgM, immunoglobulin M; IL-1β, interleukin-1β; IL-6, interleukin-6; TNF-α, tumor necrosis factor-α. Data were presented as the mean of four (ileum) and six replicates (colon) with one piglet each.

Canonical correspondence analyses (CCA) suggested TC ATTD was negatively correlated with DM, CP, OM, and EE ATTD, and serum IgG was negatively correlated with IgA, IgM, IL-6, and TNF-α (Figure 4). Ileal microbes in FSB piglets were positively correlated with CP, OM, and DM ATTD. Ileal microbes in MFSM piglets were positively correlated with TC ATTD, and FM group microbes were positively correlated with EE ATTD.

Colonic microbes in the MFSM group were positively correlated with serum IgA and IgM levels, but negatively correlated with IL-1β and TNF-α. Colonic microbes in FM animals were positively correlated with serum IgG and IL-1β, and FSB group microbes were positively correlated with IL-6 and TNF-α levels.

**FIGURE 5:**
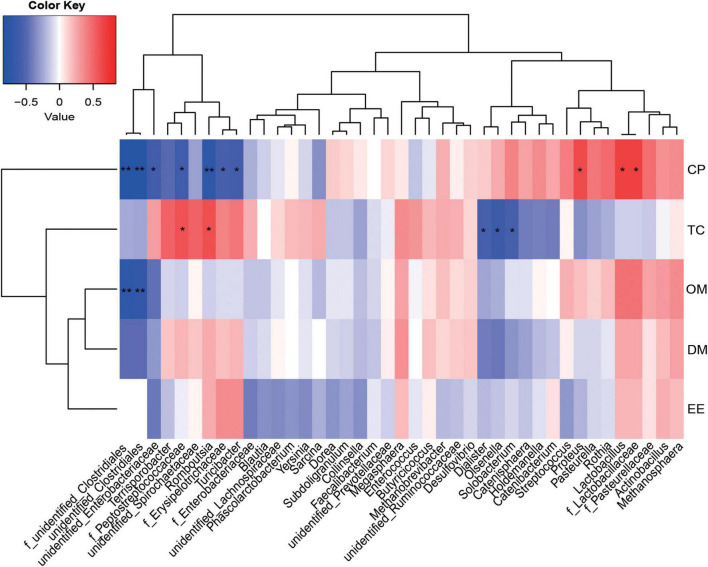
Correlations between the ileal microbiota and nutrient apparent total tract digestibility (ATTD). Red represents a positive correlation and blue represents a negative correlation. *P < 0.05 and **P < 0.01 represent significant and extremely significant correlations. DM, dry matter; CP, crude protein; EE, ether extract; OM, organic matter; TC, total carbohydrate. Data were presented as the mean of four replicates with one piglet each.

**FIGURE 6:**
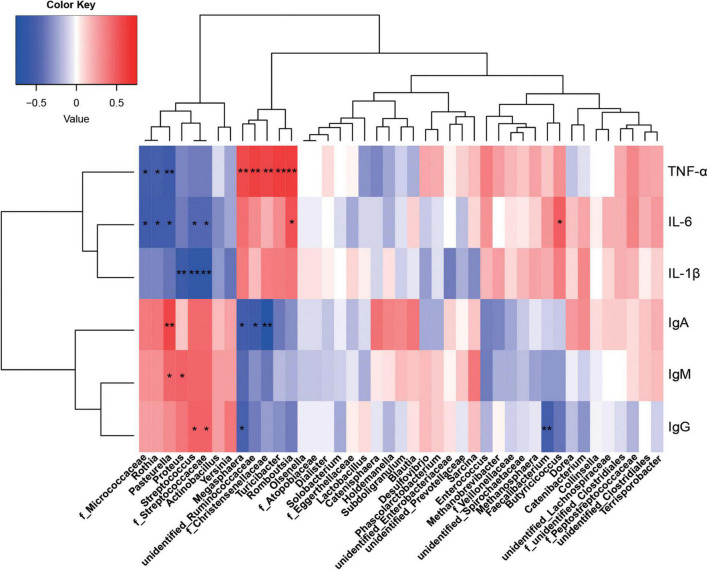
Correlations between the colonic microbiota and immune indices. Red represents a positive correlation and blue represents a negative correlation. *P < 0.05 and **P < 0.01 represent significant and extremely significant correlations. IgA, immunoglobulin A; IgG, immunoglobulin G; IgM, immunoglobulin M; IL-1β, interleukin-1β; IL-6, interleukin-6; TNF-α, tumor necrosis factor-α. Data were presented as the mean of six replicates with one piglet each.

A Spearman correlation matrix was used to explore relationships between predominant families and genera (35 most dominant genera) and ATTD or host immunity (Figures 5, 6). *F_unidentified_Clostridiales* and *unidentified_Clostridiales* were significantly negatively correlated with CP and OM ATTD (*P* < 0.05). *Unidentified_Enterobacteriaceae*, *f_Peptostreptococcaceae*, *f_Erysipelotrichaceae*, and *Romboutsia* were significantly negatively correlated with CP ATTD, while *f_Lactobacillaceae*, *Lactobacillus*, and *Proteus* were significantly positively correlated with CP ATTD (*P* < 0.05). *F_Peptostreptococcaceae* and *Romboutsia* showed significantly positive correlations with TC ATTD, but *Dialister*, *Olsenella*, and *Solobacterium* showed significantly negative correlations with TC ATTD (*P* < 0.05).

From statistical analyzes, colonic microbiota was more affected by protein sources than ileum microbiota, therefore, only correlations between colonic microbiota and immunity indices were investigated. *Megasphaera*, *unidentified_Ruminococcaceae*, and *f_Christensenellaceae* were significantly positively correlated with TNF-α, but negatively correlated with IgA (*P* < 0.05). *F_Streptococcaceae* and *Streptococcus* were significantly positively correlated with IgG, but significantly negatively correlated with IL-6 and IL-1β (*P* < 0.05). *Pasteurella* was significantly positively correlated with IgA and IgM, but negatively correlated with IL-6 and TNF-α. Also, *Proteus* was significantly positively correlated with IgM, but negatively correlated with IL-1β (*P* < 0.05). *Romboutsia* was positively correlated with IL-6 and TNF-α, *Turicibacter* positively correlated with TNF-α, and *Rothia* negatively correlated with IL-6 and TNF-α (*P* < 0.05).

### Short Chain Fatty Acid Levels in the Colon

Acetic and butyric acid levels in MFSM animals were significantly higher than levels in FSB and FM groups (*P* < 0.05; Table 6). Propionic acid levels in the MFSM group were significantly higher than those in the FSB group (*P* < 0.05). In terms of branched chain fatty acids, no significant differences were identified between groups (*P* > 0.05).

**TABLE 6:** The effects of different protein sources on colonic short chain fatty acid (SCFA) levels in weaned piglets.

Item (μMol/g)	Dietary treatment^1^			*P*-value
	FSB	FM	MFSM	
Acetic acid	48.56 ± 3.92^b^	47.75 ± 5.3^b^	56.83 ± 4.87^a^	0.02
Propionic acid	18.74 ± 1.21^b^	21.38 ± 1.16^ab^	23.54 ± 3.77^a^	0.03
Butyric acid	9.97 ± 0.63^b^	11.07 ± 2.83^b^	13.79 ± 1.59^a^	0.02
Isobutyric acid	0.91 ± 0.13	0.89 ± 0.08	0.96 ± 0.06	0.51
Isovaleric acid	1.11 ± 0.07	1.19 ± 0.06	1.28 ± 0.17	0.09
Valeric acid	2.79 ± 0.59	3.23 ± 0.85	3.76 ± 0.72	0.15

*^a,b^Different superscript letters in a row indicate a significant difference (P < 0.05).*

*^1^FSB, fermented soybean meal; FM, fish meal; MFSM, mixture of fermented soybean meal and fish meal.*

*^2^Data were the mean of five replicates (ileum) and six replicates (colon) with one piglet each.*

## Discussion

Given that high-quality protein and protein restriction can effectively decrease the amounts of proteins flowing into the hindgut, and decreased the risk of aberrant fermentation in the hindgut. Based on previous studies, no negative effects were reported on piglet growth performance when dietary CP levels were reduced by 3–4%, in contrast to the 2012 NRC recommendation of 20.5% in some piglet models (Liu et al., 2012; Gloaguen et al., 2014; Sirtori et al., 2014; Bandsma et al., 2015; Kalantar-Zadeh et al., 2016). Thus, in this study, a low-protein diet, with balanced amino acids, was used to determine interactions between dietary protein, gut microbiota, host digestibility, and health.

In our study, piglets fed a combination of FSB and FM had higher BW and ADG but a lower feed to weight gain ratio than piglets fed a single protein source, indicating piglets in the MFSM group had a higher growth rate when compared with other diets. This observation was consistent with a previous study; a combined soybean meal and fish meal diet significantly increased ADG and feed conversion ratios in piglets when compared with soybean meal or fermented soybean meal diets (Ma et al., 2019). Similarly, when compared with soy protein isolate or zein as the single dietary protein source, combination diets of soy meal, fish meal, and whey powder significantly increased piglet ADG and ADFI (Qi, 2011). A possible reason could be that ADFI was affected by different protein sources; ADFI in the FM group was significantly lower than in the FSB group during days 1–14 which may have contributed to lower growth performance. This hypothesis putatively explained our observation that piglets fed a FM diet displayed a lower growth performance than FSB-fed piglets during days 1–14. This decreased ADFI in the FM group may be related to different protein sources affecting satiety signals, as satiety induced by fish meals declined more slowly over time when compared with other protein sources (Harvey and Moore, 2004). Another possibility could be the fishy smell of fish meal, but this requires further exploration. Faster absorption rates and hormonal responses to different protein sources could also be underlying mechanisms, such as amino acids, glucagon-like peptide, and peptides derived from proteins (Boirie et al., 1997; Gustafson et al., 2001; Hall et al., 2003). Soybean meal fermentation could eliminate most anti-nutritional factors and increase the proportion of small peptides, which has great potential to improve nutrient ATTD and growth efficiency in piglets (Ma et al., 2019; Zhang and Piao, 2022). This was why CP ATTD in the FSB group was higher than in FM and MFSM groups, and why FSB-fed piglets had higher growth performance than FM-fed piglets at certain phases. However, EE ATTD in the FM group was higher than in other groups, which may explain why there was no significance in growth parameters in later phases among the three groups. Additionally, a higher piglet growth rate in the MFSM group may be attributed to the mixture of protein sources which reduced malnutrition risks and improved intestinal dysfunction (Engelsmann et al., 2022).

Most dietary nutrients are fully digested and absorbed at the end of the ileum, whereas undigested complex fragments are fermented in the hindgut (Zhang et al., 2018b). During this process, microbes promote nutrient application, and the protein sources can shape the composition of intestinal microbiota (Wu et al., 2022). The *Proteobacteria* phylum, as facultative anaerobes, cannot consume fiber, but can interfere with host nutrition by metabolizing fermentation products to carbon dioxide in the presence of oxygen (Litvak et al., 2018). As reported, the primary bacteria implicated in protein metabolism in the small intestine are: *Escherichia coli*, *Streptococcus*, *Mitsuokella*, and *Succinivibrio dextrinosolvens* (Ma et al., 2017). Piglets fed a highly digestible casein-based diet showed an increased abundance of *Enterobacteriaceae* when compared with piglets fed a soybean meal-based diet (Rist et al., 2014). Compared with degossypolized cottonseed protein, piglets fed a dried porcine protein diet showed increased intestinal *Escherichia* abundance, whereas piglets fed the degossypolized cottonseed protein diet showed a higher *Lactobacillus* abundance (Li et al., 2019a). Moreover, soy proteins and associated peptides modulated intestinal microbiota by enhancing *Lactobacilli* and *Bifidobacteria* prevalence (Huang et al., 2016). These observations were consistent with our study data; piglets fed a FM-based diet showed a higher relative abundance of ileac *unidentified_Enterobacteriaceae* and *E. coli* of the *Proteobacteria* phylum, while piglets fed FSB-based diet increased their relative abundance of *Lactobacillaceae* and *Lactobacillus* of the *Firmicutes* phylum. In addition, *unidentified_Enterobacteriaceae* negatively correlated with CP ATTD which suggested they competed with the host for nutrients and caused a lower piglet growth performance in the FM group. From previous studies, energy intake was positively associated with an increased relative abundance of *Firmicutes*, with a 20% increase in abundance associated with an additional energy harvest of 628 kJ (Hildebrandt et al., 2009; Reiner et al., 2018). These reports agreed with our data suggesting that FSB and FM combinations increased the relative abundance of *Firmicutes* and accelerated piglet growth. This positive correlation was also observed in previous piglet models (Pedersen et al., 2013; Mach et al., 2015), and in our study, was evidenced as a higher piglet growth performance in the MFSM group. Combined CCA and Spearman correlation analyzes indicated that *f_Lactobacillaceae*, *Lactobacillus*, and *Proteus* were dominant microbes in FSB animals and promoted CP digestibility, whereas *f_Peptostreptococcaceae* and *Romboutsia* were the main bacteria in MFSM animals and benefited TC digestibility. These bacteria may positively affect piglet growth performance.

Along the intestinal tract, between the ileum and colon, considerable variations in *Bacteroidetes*, *Actinobacteria*, *Firmicutes*, and *Proteobacteria* were observed. We showed that oxygen-tolerant *Proteobacteria*, such as *Enterobacteriaceae* grew well and were abundant in the ileum, but were dramatically decreased in the colon, while oxygen-sensitive *Bacteroidetes* adapting to a low oxygen environment were significantly increased in the colon. This observation agreed with a previous study showing that spatial changes in bacterial composition occurred as a result of altered microenvironments (Zhang et al., 2018b). Our colonic microbial composition data were consistent with previous findings showing that *Lactobacilaceae*, *Peptostreptococcaceae*, *Veillonellaceae*, *Ruminococcaceae*, *Clostridiales*, and *Lachnospiraceae* involving in *Firmicutes*, and *Prevotellaceae* belonging to *Bacteroidetes* were dominated taxa in the large intestine (Gill et al., 2006; Claus et al., 2011; Zhang et al., 2018b). These microbial abundance is important for complex carbohydrate digestion, amino acid biotransformation in the host.

Using excretory enzymes, *Clostridia* processes undigested dietary proteins and complex carbohydrates to produce amino acids and SCFAs (Zhu et al., 2017; Zhang et al., 2018b). Amino acids are further fermented via deamination in the colon to produce SCFAs and ammonia (Cummings and Macfarlane, 2010), which are key energy sources for colocytes. In our study, the relative abundance of *Clostridiales* in FSB and MFSM groups was higher than the FM group, while the relative abundance of *Enterobacteriaceae* was highest in the FM group when compared with the other groups. As previously reported, a host-protective mechanism against intestinal infection involves resistance between the obligate anaerobic *Clostridia* and the facultative anaerobic *Enterobacteriaceae* (Spees et al., 2013). *Clostridiales*, including *Clostridium butyrate*, stimulated immunoglobulins secretion in mice and Peyer’s patch cell (Murayama et al., 1995; Wang et al., 1996). Immunoglobulins provide passive immune protection in early life by enhancing anti-bacterial, anti-infective, and anti-viral capabilities. In our study, serum IgA on day 14 and serum IgG or IgM on day 28 in FM and MFSM groups were higher than the FSB group. Bai et al. (2016) reported that feeding soy protein and raffinose combinations markedly increased cecum IgA levels. Gut bacteria and various antigens may be involved in these changes. In our study, the relative abundance of *Streptococcus* involved in *f_Streptococcaceae* in FM-C and MFSM-C groups was significantly higher than the FSB-C group, with immunoglobulins positively correlated with *f_Streptococcaceae*, *Streptococcus*, *Pasteurella*, and *Proteus*. When combined with data from the previous study, even though *Streptococcus* is generally considered a pathogen, a modest increase in their numbers could increase IL-10 levels (Bartholomeus et al., 2014). Interleukin 10 secreted by Th2 cells stimulate B lymphocyte proliferation and generate IgG antibodies (Chang et al., 2018). These results indicated that those microbes might be the under antigens for maintaining higher immunoglobulins to protect against infection when they are in an extremely lower relative abundance.

During piglet production, weanling stress may induce intestinal disorders with increased pro-inflammatory cytokine secretion such as IL-1β, IL-6, and TNF-α, which cause growth retardation (Bomba et al., 2014; Xiong et al., 2019). Our results showed that both FM and MFSM diets significantly decreased pro-inflammatory cytokine levels when compared with the FSB diet, indicating FM alleviated host inflammatory responses in weaned piglets. This observation may be associated with microbial diversity and composition. Theoretically, highly diverse gut microbiota reflect microbial maturity, which increases with age (Turnbaugh et al., 2007). In a previous report on children, microbial diversification was a gradual process, whereas the premature formation of an adult-type microbiota negatively affected host immune function (Maynard et al., 2012; Nylund et al., 2013). This observation accorded with our study; FSB diets enhanced host inflammatory responses by increasing microbial diversity. Distinct to microbial diversity, a study showed that dietary protein sources were major factors influencing microbial composition (Rist et al., 2013). *Romboutsia* is a bacterial genus of the family *Peptostreptococcaceae*, and are generally associated with protective leukocyte antigen haplotypes in autoimmune disease (Russell et al., 2019). *Turicibacter* was shown to have a strong association with immune function, as in the intestine, *Turicibacter* was almost abolished in innate and adaptive immunodeficiency mouse models (Dorottya and Richard, 2011; Dimitriu et al., 2013). At the family level, *Christensenellaceae* was positively correlated with gut metabolites implicated in protein catabolism (Waters and Ley, 2019). In our study, these bacteria were strongly correlated with serum TNF-α and IL-6 levels, suggesting that dietary FSB-enhanced host inflammation responses may be associated with these bacteria. This was distinct to dietary FM and the combined MFSM, which increased immunoglobulin secretion.

*Clostridiales* and *Peptostreptococcus* are predominant bacteria in the large intestine of healthy humans, and they produce SCFAs and are key drivers of amino acid utilization, including lysine, threonine, proline, and glutamate (Abdallah et al., 2020). When compared with animal protein sources, soy protein intake could induce more carbohydrate metabolism and generate SCFAs (Zhu et al., 2017). The role of SCFAs in immunity is well characterized; most studies show that SCFAs sustain a balance between commensal microbes and pathogen immunity by modulating Tregs and IL-10-producing T cells, and suppressing inflammatory cytokines (Singh et al., 2014). This regulatory mechanism is associated with SCFA-G protein coupled receptors or their histone deacetylase inhibiting ability (Ara et al., 2016). These observations agreed with our data showing that a combined MFSM diet increased SCFA levels in the colon, and had lower inflammation cytokines with tolerance to *Streptococcus*, *Pasteurella*, and *Proteus*. Another reason for this could be related to tryptophan metabolism by *Peptostreptococcus* and *Clostridiales* (Abdallah et al., 2020). Indole is a predominant tryptophan metabolite which reduces severe inflammation induced by lipopolysaccharide via IL-22 up-regulation, and also enhances aryl hydrocarbon receptor activation (Shijie et al., 2018). Thus, dietary FSB and FM sources affected host health by shaping different microbiota phenotypes, and combined dietary MFSM appeared to demonstrate better tolerance to commensals.

## Conclusion

Diets containing MFSM significantly increased piglet growth performance when compared with FM-fed piglets, while diets containing FSB significantly increased CP ATTD when compared with MFSM and FM groups. Dietary MFSM and FM significantly enhanced serum immunoglobulin secretion and decreased serum cytokine production when compared with dietary FSB. Dietary FSB shaped the ileac microbiota, accelerated *f_Lactobacillaceae* and *Lactobacillus* prevalence, and increased CP digestibility, whereas dietary MFSM shaped the ileac microbiota, accelerated *f_Peptostreptococcaceae* and *Romboutsia* prevalence, and benefited TC digestibility. These microbiota phenotypes shaped by MFSM diets contributed to piglet growth performance. MFSM and FM diets also shaped a more tolerant phenotype in dominant microbiota, increasing the relative abundance of colonic *f_Streptococcaceae* and *Streptococcus* and enhancing immunoglobulin secretion. In contrast, dietary FSB shaped a more diverse microbiota phenotype, increasing the relative abundance of *Christensenellaceae* and *Romboutsia* and enhancing inflammation cytokine production. Therefore, dietary MFSM shaped particular microbiota, improving nutrient ATTD and host health which helped weaned piglets overcome weanling stress. Our study provides a theoretical basis for the application of different protein sources to the diets of young animals.

## Data Availability Statement

The datasets generated for this study can be found in the NCBI sequence read archive, accession number: PRJNA648691.

## Ethics Statement

The animal study was reviewed and approved by Institutional Animal Care and Use Committee of the Institute of Animal Science of the Chinese Academy of Agricultural Sciences.

## Author Contributions

YQ and JZ: conceptualization, methodology, and project administration. YL and YH: animal experiments, chemical analysis, and data collection. YH: writing original draft. QZ, CT, and JZ: finished writing review. All authors agreed to the final manuscript and approved the submitted version.

## Conflict of Interest

The authors declare that the research was conducted in the absence of any commercial or financial relationships that could be construed as a potential conflict of interest.

## Publisher’s Note

All claims expressed in this article are solely those of the authors and do not necessarily represent those of their affiliated organizations, or those of the publisher, the editors and the reviewers. Any product that may be evaluated in this article, or claim that may be made by its manufacturer, is not guaranteed or endorsed by the publisher.

## Footnotes

1
http://ccb.jhu.edu/software/FLASH/

2
http://drive5.com/uparse/

3
http://www.arb-silva.de/

4
http://www.mothur.org/wiki/Chao

5
http://www.mothur.org/wiki/Ace

6
http://www.mothur.org/wiki/Shannon

7
http://www.mothur.org/wiki/Simpson
